# Unprecedented continental drying, shrinking freshwater availability, and increasing land contributions to sea level rise

**DOI:** 10.1126/sciadv.adx0298

**Published:** 2025-07-25

**Authors:** Hrishikesh A. Chandanpurkar, James S. Famiglietti, Kaushik Gopalan, David N. Wiese, Yoshihide Wada, Kaoru Kakinuma, John T. Reager, Fan Zhang

**Affiliations:** ^1^ FLAME University, Pune, India.; ^2^ School of Sustainability, Arizona State University, Tempe, AZ, USA.; ^3^ Jet Propulsion Laboratory, California Institute of Technology, Pasadena, CA, USA.; ^4^ Biological and Environmental Science and Engineering Division, King Abdullah University of Science and Technology, Thuwal, Saudi Arabia.; ^5^ Moon Soul Graduate School of Future Strategy, Korea Advanced Institute of Science and Technology, Daejeon, Republic of Korea.; ^6^ The World Bank, Washington, DC, USA.

## Abstract

Changes in terrestrial water storage (TWS) are a critical indicator of freshwater availability. We use NASA GRACE/GRACE-FO data to show that the continents have undergone unprecedented TWS loss since 2002. Areas experiencing drying increased by twice the size of California annually, creating “mega-drying” regions across the Northern Hemisphere. While most of the world’s dry/wet areas continue to get drier/wetter, dry areas are now drying faster than wet areas are wetting. Changes in TWS are driven by high-latitude water losses, intense Central American/European droughts, and groundwater depletion, which accounts for 68% of TWS loss over non-glaciated continental regions. “Continental drying” is having profound global impacts. Since 2002, 75% of the population lives in 101 countries that have been losing freshwater water. Furthermore, the continents now contribute more freshwater to sea level rise than the ice sheets, and drying regions now contribute more than land glaciers and ice caps. Urgent action is required to prepare for the major impacts of results presented.

## Teaser

Drying continents, extreme drought, and groundwater depletion are shrinking water availability and increasing sea level rise.

## INTRODUCTION

Climate change is driving profound changes within the Earth system, including to its water cycle. While global temperatures continue to reach record heights (*1*–*3*), with the year 2024 being the hottest year in the past 175 years (*4*), the planet is experiencing increasing extremes of flooding and drought (*5*), widespread glacial and ice sheet melt and sea level rise (*6*–*8*), and greater risk of wildfire (*9*) and biodiversity loss (*10*).

As global patterns of precipitation, evaporation, and streamflow change (*11*), terrestrial water storage (TWS; all of the ice, snow, surface water, canopy water, soil moisture, and groundwater stored on land) has been shifting rapidly in response (*12*–*15*). Shifting patterns of TWS threaten water availability and sustainable water management for people and the environment, putting livelihoods and food security at risk (*16*) while acting as a trigger for climate migration (*17*) and transboundary conflict (*18*) both intra- and internationally.

As the dry areas of the world become drier (*13*, *16*, *19*) and surface water storage in rivers and lakes declines (*12*), society is becoming more reliant on groundwater (*20*). This increased reliance has led to its long-term depletion (*21*, *22*), which is exacerbated by global shortcomings in groundwater management (*20*, *23*) and which amplifies rates of TWS loss through a positive feedback. The consequences of global groundwater depletion include reduced irrigation water supply and threats to agricultural productivity, reduced capacity for climate adaptation, drought resilience and for growth in desert cities, reduced biodiversity (*24*) and damage to groundwater dependent ecosystems (*25*), decreasing access as water tables fall, and many others (*21*, *26*, *27*).

Global TWS changes also have major consequences for interannual variations in sea level and long-term global mean sea level (GMSL) rise (*28*–*30*). The loss of freshwater from the continents and the ice sheets eventually leads to a corresponding increase in ocean water mass. While the continental contribution drives GMSL variations at seasonal and interannual timescales (*28*), its long-term contribution at longer timescales has, until recently (*8*), been smaller than the ice sheets on human timescales. If the TWS trends identified here continue, then this may never again be the case.

Here, we use more than two decades of observations (April 2002 to April 2024) from NASA’s Gravity Recovery and Climate Experiment (GRACE) and GRACE Follow-On (GRACE-FO) missions (hereafter, GRACE/FO) (*31*, *32*) to evaluate how and why TWS has changed since 2002. We find that the continents (all land excluding Greenland and Antarctica) have undergone unprecedented rates of drying and that the continental areas experiencing drying are increasing by about twice the size of the State of California each year. The rapid expansion of dry areas has resulted in the emergence of “mega-drying” regions by interlinking of previously known drying hot spots (*13*), particularly since the strongest recorded El Niño of 2014 and across the Northern Hemisphere.

We find that, while most of the world’s dry areas continue to get drier and its wet areas continue to get wetter, dry areas are drying at a faster rate than wet areas are wetting. At the same time, the area experiencing drying has increased, while the area experiencing wetting has decreased. We show that changes in TWS since previous global studies (*13*, *19*) are robust and are driven primarily by high-latitude water losses in Canada and Russia (most likely due to ice and permafrost melt), by the extreme Central American and European droughts of the past several years (*33*), and by continued global groundwater depletion (*13*, *14*, *21*, *22*), which accounts for 68% of the TWS trend over the non-glaciated continents.

We refer to the phenomenon of global-scale reduction in TWS as measured by the GRACE/FO missions as “continental drying.” Our definition of continental drying implicitly includes melting of glaciers and ice caps (GICs) on land (distinct from the Greenland and Antarctic ice sheets, see fig. S1). However, the expansion of drying regions reported here occurs in the non-glaciated regions. We take care to differentiate in the analyses below.

Here, we explore current rates of continental drying and its regional contributors, we discuss the implications of our findings by demonstrating their impact on freshwater availability and sea level rise, and we attempt to attribute its causes to human water management practices (e.g., overpumping groundwater) and/or to changing climate to help support evidence-based policy and decision-making.

We note that this continental drying affects most of the population and countries in the world. Roughly 75% of the global population lives in the 101 countries that have been losing freshwater water since 2002. Within the global water budget, the continents are now contributing more freshwater to sea level rise than the individual ice sheets. Furthermore, the drying regions on the continents now contribute more to sea level rise than GICs on land. Without urgent attention and action, the findings presented here may well continue to worsen, leading to accelerations in water insecurity (*20*, *23*, *26*, *27*) and sea level rise (*29*, *30*, *34*).

## RESULTS

### The emergence of mega-drying regions on the continents

**Fig. 1.**
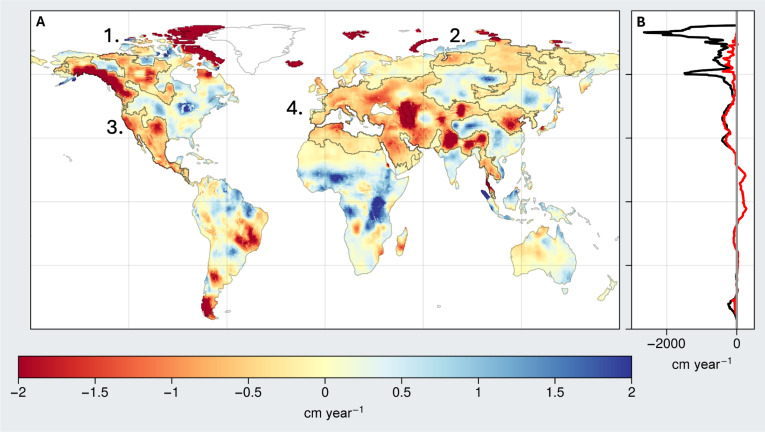
Global map of long-term TWS trends from GRACE/FO. (**A**) Trends in TWS (cm year^−1^) from February 2003 to April 2024 (see Materials and Methods). Mega-regions (regions exceeding −0.2 cm year^−1^ and connecting previously reported TWS hot spots) are outlined in black and labeled 1 to 4 corresponding to the main text. (**B**) Zonal sum of TWS trends for all (black) and non-glaciated regions (red).

Previous studies have documented regional and global patterns of TWS change (*13*, *14*, *19*) like those shown in the bias-corrected (i.e., downscaled) GRACE/FO trend map (see Materials and Methods) in Fig. 1B. These studies have identified the key features of TWS change on the continents, including high- and low-latitude wet areas getting wetter (WW) and mid-latitude dry areas getting drier (DD) as anticipated from climate change modeling studies (*35*), glacier and ice cap melting (*7*), global-scale groundwater depletion (*13*, *14*), and changing extremes of flooding and drought (*5*). Here, we show how recent changes in these regional and continental-scale TWS trend patterns are contributing to the increasing rates of continental drying reported in this study.

A critical, major development has been the interconnection of several regional drying patterns and previously identified hot spots for TWS loss to form four continental-scale mega-drying regions, all located in the Northern Hemisphere (shown with solid black outlines in Fig. 1B). These include (i) large swaths of northern Canada and (ii) northern Russia, where high-latitude wetting has now transitioned to drying; (iii) the contiguous region of southwestern North America and Central America, where aridification and groundwater depletion continue or are worsening; and (iv) the massive, tri-continental region spanning from North Africa to Europe, through the Middle East and Central Asia, to northern China and South and Southeast Asia, which owes its expansion to the recent European drought (*33*).

These changes, along with the more recent pronounced wetting of East Africa and western Sub-Saharan Africa, underpin the finding of expanding drying continental regions and shrinking wetting regions reported here. They are also reflected in the zonal plot in Fig. 1B. Except for the tropics between 10°S and 20°N, all latitudes now show a net negative TWS trend, even when excluding continental GICs. This result deviates from past studies (*13*, *19*) that documented first TWS observations of “wet getting wetter, dry getting dryer” (WW-DD), showing an increasing TWS trend in the Northern high latitudes. All trends discussed in this study are summarized in Tables 1 to 3 and table S1. As discussed below, we find that the trends reported here are robust and, therefore, unlikely to change, rather than emerging, as in previous studies [e.g., (*13*)].

**Table 1.** Long-term TWS trends in mega-drying regions from GRACE/FO. Trends (cm year^−1^) are from February 2003 to April 2024 and are given with and without continental GICs when they are present. Uncertainties represent 90% confidence intervals on the trend estimation. AZ, Arizona; CA, California; CO, Colorado; KS, Kansas; NM, New Mexico; NV, Nevada; OK, Oklahoma; TX, Texas; UT, Utah.

Mega-drying regions	TWS trend in cm year^−1^
**Northern Canada and Alaska**	−0.86 ± 0.03
Northern Canada and Alaska with GICs	−2.23 ± 0.05
**Northern Russia**	−0.41 ± 0.03
Northern Russia with GICs	−0.42 ± 0.03
**Southwestern North America and Central America**	−0.76 ± 0.04
Southwestern US states (AZ, CA, CO, KS, NM, NV, OK, TX, and UT combined)	−0.85 ± 0.05
Mexico and Central America combined	−0.66 ± 0.05
**MENA and Pan-Eurasia**	−0.83 ± 0.02
MENA and Pan-Eurasia with GICs	−0.88 ± 0.02
Northwest Sahara Aquifer System	−0.45 ± 0.01
Arabian Aquifer System	−0.64 ± 0.01
Caspian and Aral Seas	−3.0 ± 0.12
Tarim Basin	−0.39 ± 0.01
Tarim Basin with GICs	−0.52 ± 0.02
Indus Basin	−1.23 ± 0.07
Ganges-Brahmaputra Basin	−1.09 ± 0.09
Ganges-Brahmaputra Basin with GICs	−1.4 ± 0.09
North China Aquifer System	−0.82 ± 0.1
Myanmar	−0.37 ± 0.09
Thailand	−0.94 ± 0.1
Cambodia	−0.54 ± 0.13
Malaysia	−0.6 ± 0.06

**Table 2.** Rates of expansion of areas experiencing drying and wetting, including extremes. The rates are for the period from February 2004 to April 2024 unless noted otherwise. Both areas with and without GICs are mentioned. Uncertainties represent 90% confidence intervals on trend estimation.

Areas under drying	km^2^ year^−1^
Total land areas under dry anomalies	831,600 ± 69,100
Total land areas excluding GICs under dry anomalies	601,500 ± 65,200
Total land areas under dry extremes	845,065 ± 122,661
Total land areas excluding GICs under dry extremes	685,096 ± 110,021
Total land areas excluding GICs under dry extremes (February 2002 to December 2013)	−706,800 ± 156,500
Total land areas excluding GICs under dry extremes (January 2014 to April 2024)	2,610,000 ± 242,900
**Areas under wetting**	**km**^**2**^ **year**^**−1**^
Total land areas under wet anomalies	−831,600 ± 69,100
Total land areas excluding GICs under wet anomalies	−601,500 ± 65,200
Total land areas under wet extremes	−232,300 ± 128,800
Total land areas excluding GICs under wet extremes	−113,700 ± 117,000
Total land areas excluding GICs under wet extremes (February 2002 to December 2013)	−1,847,100 ± 226,600
Total land areas excluding GICs under wet extremes (January 2014 to April 2024)	1,650,900 ± 127,200

**Table 3.** Contributions to sea level rise from major global water reservoirs. Trend in mm SLE year^−1^ and Gt year^−1^ or km^3^ year^−1^. Includes decomposition of non-glaciated TWS into drying and wetting regions. Positive trends increase sea level and negative trends decrease sea level.

Global water reservoirs	Trend values (mm SLE year^−1^)	Trend values (Gt or km^3^ year^−1^)
Global ocean	1.99 ± 0.2	724 ± 69
Greenland	0.73 ± 0.07	266 ± 25
Antarctica	0.37 ± 0.05	135 ± 19
Global land (TWS)	0.89 ± 0.15	324 ± 55
GICs	0.67 ± 0.04	243 ± 14
Non-glaciated TWS	0.22 ± 0.14	81 ± 52
**Drying regions**		
Non-glaciated TWS drying regions	1.01 ± 0.11	368 ± 40
Robust drying regions	1.29 ± 0.1	467 ± 37
Non-glaciated robust drying regions	0.7 ± 0.12	260 ± 43
**Wetting regions**		
Non-glaciated TWS wetting regions	−0.79 ± 0.12	−287 ± 44
Robust wetting regions	−0.44 ± 0.11	−161 ± 41
Non-glaciated robust wetting regions	−0.43 ± 0.11	−153 ± 41

#### Are high-latitude wet areas still getting wetter?

While glaciers melting in coastal Alaska and coastal western Canada and ice cap melting in the Canadian Archipelagos have long contributed to high-latitude TWS decline (*8*, *36*, *37*), the remaining non-glaciated high-latitude continents had been mostly increasing in TWS, largely driven by WW (*13*, *19*). However, as high latitudes warm at four times the global average rate (*38*), interior western Canada is now losing TWS likely due to drying of subarctic lakes (*12*), the Canadian prairies have experienced persistent drought for the past several years (*39*), and TWS is declining in northern Russia due to changes in precipitation and potentially due to changes in permafrost (*40*, *41*). Excluding GIC, the northern Canada mega-region TWS trend is −0.86 ± 0.03 cm year^−1^, and the northern Russia trend is −0.41 ± 0.03 cm year^−1^. Widespread but more dispersed drying is also seen across northern Europe/Scandinavia. While the atmospheric mechanisms of WW-DD are not disputed, it is possible that Coupled Model Intercomparison Project (CMIP) models (*42*, *43*) do not adequately represent ice, snow, and permafrost melt and declining surface water storage and, hence, cannot capture the emerging dynamics of TWS drying at high latitudes.

#### Southwestern North America and Central America

Several studies have focused on hot spots for decreasing TWS in the southwestern quadrant of the United States (−0.85 ± 0.05 cm year^−1^), especially due to groundwater depletion in California’s Central Valley (*44*–*46*) and the southern Ogallala Aquifer of the US High Plains (*45*), aridification (*47*), and groundwater depletion in the Colorado River basin (*48*, *49*). In contrast to earlier reports (*13*, *50*) that showed near-zero or wetting TWS trends in Mexico and Central America, we show here that they are undergoing recent and rapid TWS decline (−0.66 ± 0.05 cm year^−1^). Furthermore, its extent now links together with California, the lower Colorado River basin, and the southern High Plains to create one large southwestern North American–Central American mega-drying region. This region includes the well-documented areas for groundwater depletion noted above, as well as in Mexico City (*51*), that are all exacerbated by DD and ongoing drought (*33*). The rate of declining TWS across the entire mega-region is −0.76 ± 0.04 cm year^−1^.

#### *Middle East/North Africa*–*Pan-Eurasia*

The outline in Fig. 1B shows the tremendous extent of this mega-drying region, which is losing TWS at the rate of −0.88 ± 0.02 cm year^−1^. The region is dominated by DD, which places even more stress on its dwindling groundwater resources. In contrast to earlier findings (*13*, *19*), this study finds a recent pronounced decline in TWS across much of Europe, consistent with the recent catastrophic drought events that were found to be influenced by climate change and are among the worst in the past 2000 years (*52*). The drying now includes the British Isles and all the countries in Western and Eastern Europe. North Africa almost entirely shows a decline due to DD (*3*, *52*), as well as from considerable groundwater depletion in the North-Western Sahara Aquifer System shared by Algeria, Libya, and Tunisia (*14*, *53*).

Pronounced drying across the Middle East through DD and groundwater depletion has been well-documented (*14*, *54*, *55*) and continues to be among the most severe in the world. Here, we highlight trends in this mega-region that have been relatively underreported in the literature. For example, the Arabian Aquifer System (*56*, *57*) is losing TWS at a rate of 0.64 ± 0.01 cm year ^−1^. Central Asia is rapidly losing TWS through DD and groundwater depletion, particularly around the Caspian and Aral seas, where agriculture and cotton production rely heavily on groundwater (*58*, *59*). TWS losses in the combined Caspian (*60*) and Aral Seas are −3.0 ± 0.12 cm year^−1^.

Groundwater depletion continues unabated in the agricultural regions on the perimeter of the Tibetan Plateau. Excluding GIC, TWS losses in the Tarim Basin are −0.39 ± 0.01 cm year^−1^, in the Indus Basin are −1.23 ± 0.07 cm year^−1^, and in Ganges-Brahmaputra Basin (*61*) are −1.09 ± 0.09 cm year^−1^ [which are consistent/worse than previous reports (*12*, *13*, *61*–*63*)]. The extent of the tri-continental drying region now links to the North China Aquifer System (*64*) to the east, where groundwater depletion is driving TWS declines to −0.82 ± 0.1 cm year^−1^, and to Southeast Asia, where DD is now responsible for declines in Myanmar (−0.37 ± 0.09 cm year^−1^), Thailand (−0.94 ± 0.1 cm year^−1^), Cambodia (−0.54 ± 0.13 cm year^−1^), and Malaysia (−0.6 ± 0.06 cm year^−1^).

#### Increase in the land areas under drying and dry extremes

**Fig. 2.**
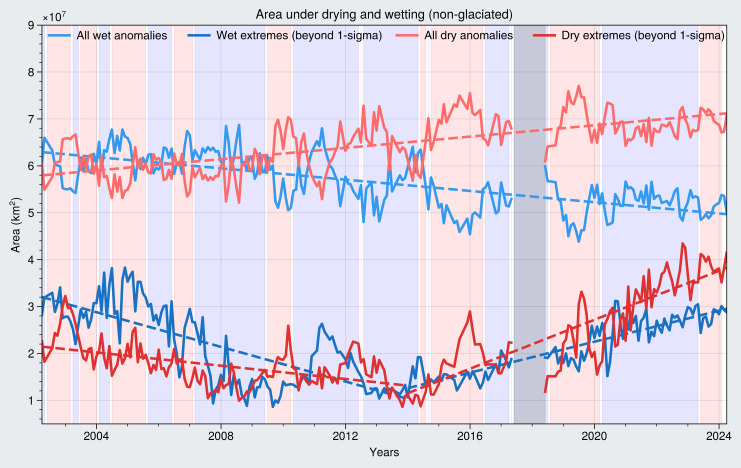
Changes in areas experiencing wet and dry conditions, including extremes. Changes in the global non-glaciated area under dry (light red) and wet (light blue) anomalies (monthly deviations from monthly climatology) and under dry (dark red) and wet (dark blue) extremes (monthly anomalies that are greater than 1 SD at each location). Positive (La Niña) and negative (El Niño) ENSO events shown as blue and red background color, respectively. Vertical gray bar represents gap between GRACE and GRACE-FO.

The emergence of interconnected mega-drying regions is coincident with the increase in area on the continents showing monthly dry anomalies. The rate of increase of drying areas is 831,600 ± 69,100 km^2^ year^−1^, which is roughly equivalent to twice the size of the State of California annually. The area experiencing dry extremes (defined here as dry anomalies that are greater than 1-sigma, see Material and Methods) also grew by a similar rate of 845,000 ± 122,600 km^2^ year^−1^. These increases are largely driven by drying in the non-glaciated regions (Fig. 2), where the area under dry anomalies increased by 601,500 ± 65,200 km^2^ year^−1^ and area under dry extremes increased by 685,100 ± 110,000 km^2^ year^−1^.

Since 2014, the year of onset of the strongest El Niño on record, the total non-glaciated area experiencing extreme drying began increasing rapidly, by 2,610,000 ± 242,900 km^2^ year^−1^. This is despite the fact that recent years have been dominated by La Niña events (blue background in Fig. 2) and that La Niña events typically result in above average TWS (*29*, *30*).

Complementing the increased drying, both the area that is getting wetter and the area that is experiencing wet extremes have decreased (Fig. 2). The wet areas decreased by −831,600 ± 69,100 km^2^ year^−1^ (and by 601,500 ± 65,200 km^2^ year^−1^ over non-glaciated land). These values are the inverse of areas under drying, see Material and Methods), while the area experiencing wet extremes decreased by −232,300 ± 128,800 km^2^ year^−1^. However, as in the case of the drying extremes, growth in the area experiencing wet extremes begins increasing around 2014, but at a slower rate (1,650,900 ± 127,200 km^2^ year^−1^ over non-glaciated land) than that of the areas experiencing dry extremes.

**Fig. 3.**
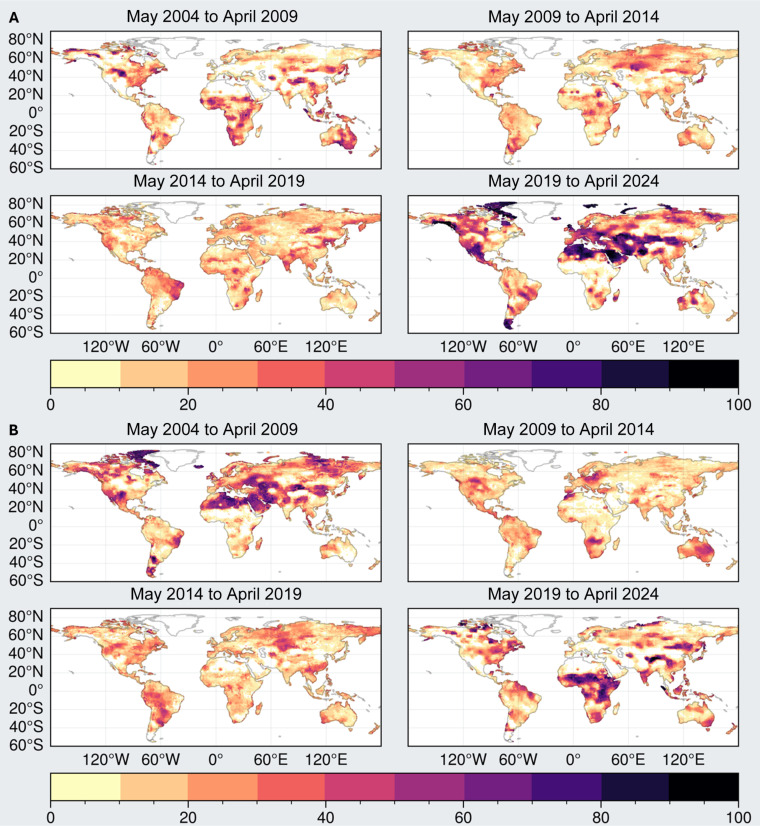
Mapping location and presence of dry and wet extremes. (**A**) Percent of months in successive 5-year periods for which a region experienced dry extremes. (**B**) As in (A) but for extreme wetting. “Extremes” here are defined as monthly TWS anomalies at each location that are greater than 1-sigma (1 SD of the local deseasonalized TWS).

Figure 3 shows how the dry and wet extremes have changed in location and existence in the past 20 years, in successive 5-year periods (May 2004 to April 2009, May 2009 to April 2014, May 2014 to April 2019, and May 2019 to April 2024). Specifically, Fig. 3A shows how the percentage of months experiencing extreme drying decreases through April 2014 and then begins increasing in May 2014 (see figs. S2 and S3 for yearly maps of the number of months each year of extreme drying/extreme wetting anomalies). The final 5-year period shows a marked increase in the percentage of months under dry extremes, that the spatial distribution of the affected regions closely corresponds with the shapes of the mega-drying regions discussed above, and a shift from most dry extremes occurring in the southern hemisphere to most occurring in the Northern Hemisphere and for a longer duration. Hence, it is clear that increasing extremes of drought, in both areas, location and duration, are driving the growth of previously identified hot spots or drying regions, into interconnected, continental-scale mega-drying regions, particularly in the Northern Hemisphere.

The corresponding changes in extreme wetting area are shown in Fig. 3B. As above, they complement the changes in extreme drying area, in both timing and in location, from predominantly occurring in the Northern Hemisphere before May 2014 to predominantly in the southern hemisphere by the past 5-year period.

To our knowledge, this hemispheric oscillation in TWS has not been documented. It may well be tied to longer-term oscillations like the Pacific Decadal Oscillation (PDO). Previous research suggests the PDO has a greater influence on TWS distributions than El Niño–Southern Oscillation (ENSO) at decadal timescales (*65*–*67*).

#### Robustness of trends and implications for shrinking freshwater availability

**Fig. 4.**
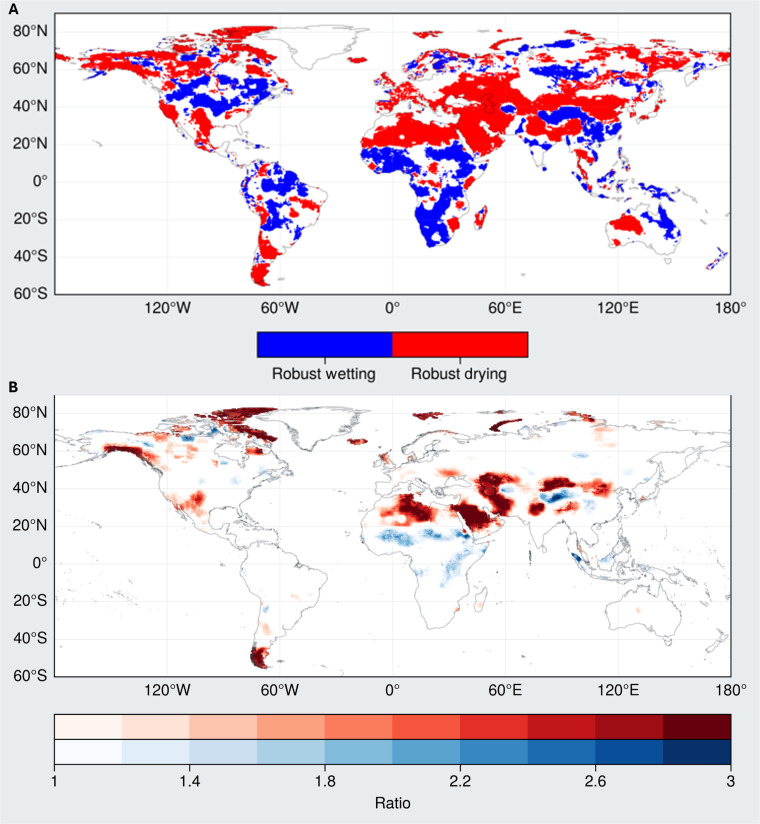
Mapping robustness of TWS trends. (**A**) Drying and wetting land regions from Fig. 1B where the TWS trend sign has been persistent and less sensitive to the increasing GRACE/FO record length. (**B**) Ratio of local interannual variability of detrended TWS anomalies to their long-term local trends. The red and blue color bars indicate regions with decreasing TWS trend and increasing TWS trend from Fig. 1B, respectively.

Here, we discuss the spatial trends in terms of their robustness and long-term persistence relative to interannual variability. The regions highlighted in Fig. 4A are those where the sign of the local trends has remained the same for more than 90% of iterations of increasing GRACE/FO record length (see Materials and Methods). These regions show reduced sensitivity to the increasing data records, i.e., they have been showing reliable long-term trends for the past 22 years, which arguably can be expected to persist in near future.

An important finding is that most of the drying regions discussed in the previous section show little sensitivity to a lengthening GRACE/FO record. In other words, their drying is robust. These include most of the continental GIC regions (e.g., Alaska, the Canadian Archipelago, and Patagonia), much of the Middle East/North Africa (MENA)/Pan-Eurasia mega-drying region, the high-latitude Canada and northern Eurasia mega-drying regions, and several of the world’s major aquifer systems discussed above and listed in Table 1 and table S1 and shown in fig. S4 (e.g., California’s Central Valley; southern Ogallala Aquifer; Northwestern Sahara Aquifer System; Arabian Aquifer System; the Tarim, Indus, and Ganges-Brahmaputra Basins; and North China Aquifer System). On the other hand, most of the tropical wetting regions (e.g., eastern Brazil, Sub-Saharan and Rift Valley Africa, central India, Indonesia, and the South Pacific Islands of Oceana) also show little sensitivity, as do pockets of high-latitude wetting on all the continents. However, the majority of the area under persistent trends is drying (62%).

Further insight into the relative importance of the long-term trends compared to interannual variability is provided in Fig. 4B. The values represent the ratio between long-term variance and the interannual variance. Long-term variance is represented as the SD of the linear fit to the long-term trend, and the interannual variance is represented as the SD of the TWS anomalies after removing the long-term trend. A ratio greater than 1 indicates that the variance from the long-term trend is at least as big as that from the interannual variability. In line with the findings of the above sensitivity test, an overwhelming number of locations (73%) where the long-term variance is more than interannual variance are in the drying regions and have long-term variance to interannual variance ratio as high as 5.8.

In contrast, trends in several locations that show prominent wetting in Fig. 4B, such as in eastern North America, northeastern South America, southern Africa, and eastern Australia, are less than the interannual variability experienced by these regions. While some of these also show up as robust in the above sensitivity test, they have been dominated by some pronounced wet extreme events (*29*, *65*, *66*) and, consequently, their persistence is uncertain.

The locations where the spatial TWS trends are persistent and where the long-term variance exceeds the interannual variance are best understood in the context of their geography and characteristics. Land GICs are anchored in space and are continuously melting in response to rising temperatures. The progression of some of the high-latitude regions from WW to drying reflects the rapidly warming environment and the long-term melting of ice and permafrost and which contribute to the disappearance of subarctic lakes. Groundwater aquifers are fixed locations that are being heavily exploited for irrigated agriculture. Mid-latitude DD regions already have low annual rainfall totals that are expected to decrease, while evapotranspiration rates are increasing. In the absence of major climate change and water management interventions, these drying TWS trends are expected to continue. Likewise, tropical regions have a strong relationship to ENSO and other climate variability modes, have a pronounced annual cycle, and are wet regions to begin with. While some of these might follow the long-term WW phenomena, the dominance of interannual variability along with the modulation of the pronounced seasonal cycle (*28*, *65*–*67*) is likely to continue.

### Shrinking freshwater availability

**Fig. 5.**
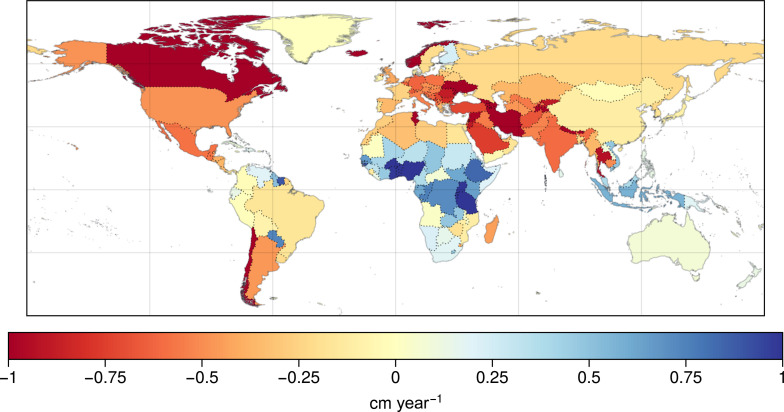
Long-term mean TWS trends from GRACE/FO by country. TWS trends (February 2003 to April 2024) averaged for every country.

The implications of continental drying for freshwater availability are potentially staggering. Nearly 6 billion people, roughly 75% of world’s population in 2020, live in the 101 countries that have been losing freshwater over the past 22 years (Fig. 5). Key contributors to the expansion of drying regions, declining TWS, and shrinking freshwater supply include melting GICs, the increasing severity of drought, the decreasing surface water availability, and groundwater depletion and all are continuing. Recent studies estimate that up to 83% of world’s glaciers will likely melt out over the next 80 years (*68*); that the severity of drought has worsened in the past 5 years (*4*, *5*, *30*, *33*, *52*); that surface water storage in rivers, lakes, and reservoirs is in decline (*11*); and that half of the world’s major aquifers are being rapidly depleted (*13*, *14*, *26*, *27*, *50*).

To assess the importance of TWS trends at the local level, we compared the TWS trends with annual renewable freshwater for level 5 HydroBASINS (*69*). The annual renewable freshwater is defined as precipitation minus evapotranspiration minus the environmental flow requirement (*70*). As illustrated in fig. S5, the median long-term TWS trend magnitude is about 3% of annual freshwater supply for all basins, 5% for the basins that are drying, and 2% for the basins that are wetting. The importance of the median TWS trend increases for basins that are arid (8%) and basins that are arid and experiencing drying (10%). The basins highlighted in red indicate regions where water losses and demands have persistently outpaced renewable supply, making it increasingly difficult to offset water storage deficits (*14*).

We used a global hydrological model [WaterGAP2.2d (*71*); see Materials and Methods] to show that the largest single contributor (68%) to the TWS loss on the non-GIC drying continental areas comes from groundwater (*29*), followed by surface water (18%), soil moisture (9%), and snow water equivalent (5%) (see figs. S1 and S6). Given these current trajectories, rates of continental drying are on track to continue or increase in the coming decades, and, consequently, freshwater availability will continue its current or accelerated decline.

### Contributions to sea level rise

**Fig. 6.**
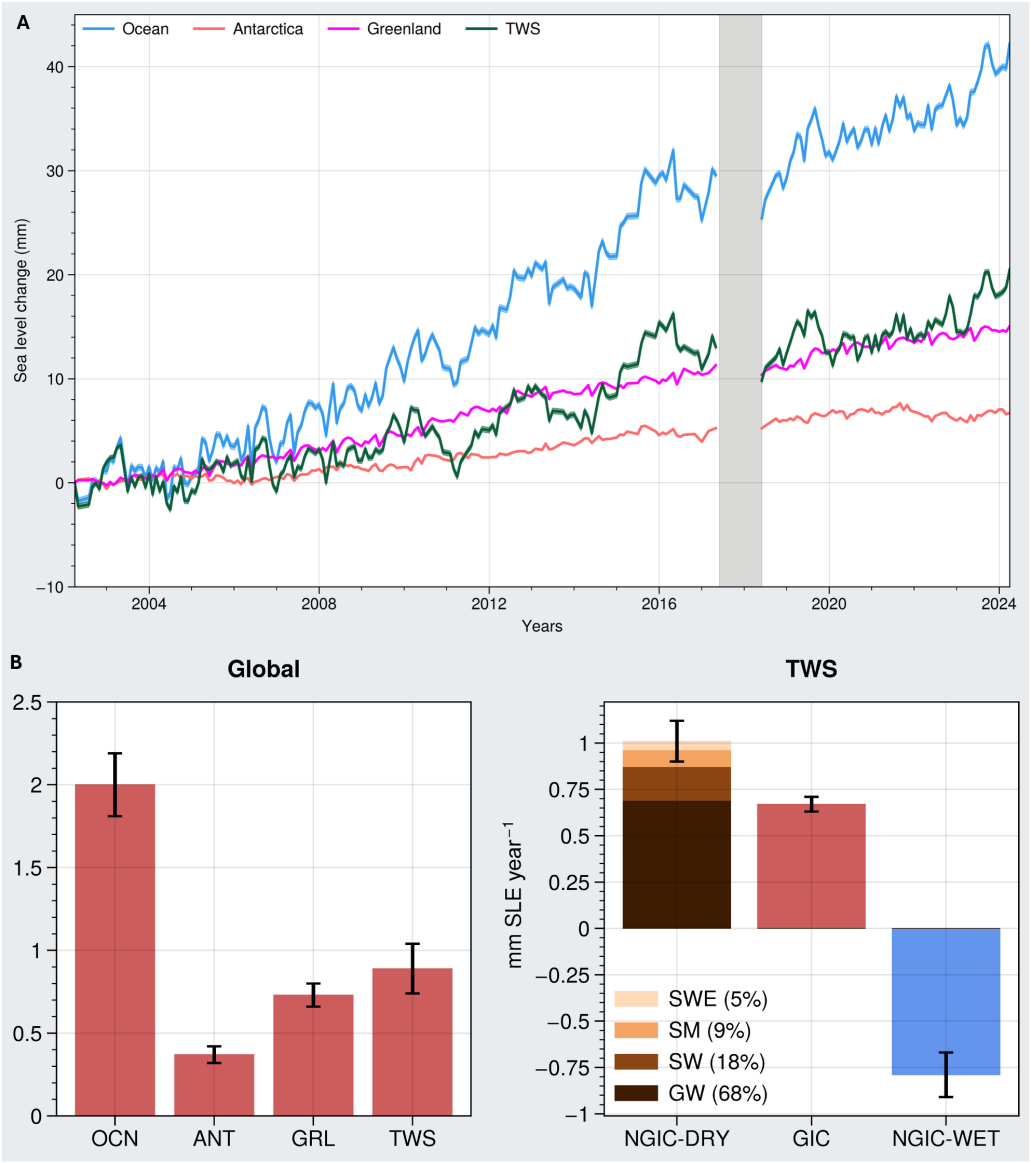
Global water mass contributions to sea level rise. (**A**) Time series of water mass anomalies of the major global water reservoirs (Ocean, Antarctica, Greenland, and TWS on the continents) from GRACE/FO from April 2002 to April 2024, in mm SLE. (**B**) Left: Corresponding trends (in mm SLE year^−1^) for the ocean (OCN), Antarctica (ANT), Greenland (GRL), and TWS time series in (A). Water mass contributions that increase sea level are shown as positive. Right: TWS trend decomposed into its non-glaciated drying (NGIC-DRY), glaciated drying (GIC), and non-glaciated wetting (NGIC-WET) components. NGIC-DRY is further decomposed into groundwater (GW), surface water (SW), soil moisture (SM), and snow water equivalent (SWE), on the basis of their contributions to the TWS trend. See also figs. S1 and S6.

We present the latest GRACE/FO water mass anomaly time series for the global ocean, the Greenland and Antarctic ice sheets, and the continents (Fig. 6A), including their secular trends over the 22 years of GRACE/FO observations (Fig. 6B, left), also summarized in Table 3. Note that, in Fig. 6 and Table 3, decreasing (drying) trends in TWS are positive contributions to GMSL, and increasing (wetting) trends in TWS are negative contributions to GMSL. Global ocean mass continues to increase at a rate of 1.99 ± 0.2 mm Sea Level Equivalent (SLE) year^−1^. Figure 6A shows that, since 2015, it is being driven primarily by decreasing TWS (0.89 ± 0.15 mm SLE year^−1^), rather than by the melting of the Greenland (0.73 ± 0.07 mm SLE year^−1^) and Antarctic (0.37 ± 0.05 mm SLE year^−1^) ice sheets. In other words, the continents are now the leading contributor (44%) to mass-driven GMSL rise, while Greenland and Antarctica contribute ~37 and ~19% respectively.

To better understand the dynamics of continental drying and their contributions to GMSL, we decompose the continents into their drying and wetting components (Fig. 6B, right; see Materials and Methods and fig. S1) using the global map of TWS trends in Fig. 1B and the global hydrological model mentioned above (see also fig. S6). Consistent with earlier work (*19*), we find that pronounced drying signal (1.7 mm SLE year^−1^) observed on the continents gets somewhat dampened by the wetting signal (−0.81 mm SLE year^−1^) due to strong, climate-driven wetting trends in a few key regions mentioned previously.

However, in the drying regions, melting of land GICs (0.67 mm SLE year^−1^), i.e., GIC, is no longer the leading continental contribution to GMSL rise. Rather, it is now outpaced by the combination of increasing drought and increasing groundwater depletion in the remainder of the drying regions of the continents (1.01 mm SLE year^−1^; NGIC-DRY in Fig. 6B, right). As discussed above, changing extremes are an important contributor to the expansion of continental drying beyond the glaciated regions, and hence to rates of GMSL rise. However, most of these non-glaciated drying trends are robust (based on the trend persistence test illustrated in Fig. 4A), contributing 0.7 mm SLE year^−1^ to GMSL, while having about 40% lower year-to-year variability compared to robust non-glaciated wetting regions (fig. S7, A and B).

## DISCUSSION

The expansion of continental drying, the increase in extreme drying, and the implications for shrinking freshwater availability and sea level rise should be of paramount concern to the general public, to resource managers, and to decision-makers around the world. The robustness of the trends reported here, along with a critical shift in the behavior of TWS and continental drying following the major El Niño beginning in 2014, may well mean that reversing these trends is unlikely. Combined, they send perhaps the direst message on the impact of climate change to date. The continents are drying, freshwater availability is shrinking, and sea level rise is accelerating (*34*).

### Implications for freshwater availability

At present, overpumping groundwater is the largest contributor to rates of TWS decline in drying regions, significantly amplifying the impacts of increasing temperature, aridification, and extreme drought events. However, groundwater depletion is most directly affected by, and can also be arrested by, water management decisions. The continued overuse of groundwater, which, in some regions like California, is occurring at an increasing, rather than at sustainable or decreasing rates (*46*), undermines regional and global water and food security in ways that are not fully acknowledged around the world.

In many places where groundwater is being depleted, it will not be replenished on human timescales. The disappearance of groundwater from the world’s aquifers (*13*, *14*, *20*, *26*, *27*, *50*) is a critical, emerging threat to humanity and presents cascading risks that are rarely incorporated in environmental policy, management, and governance. It is an intergenerational resource that is being poorly managed, if managed at all (*20*, *26*, *50*), by recent generations, at tremendous and exceptionally undervalued cost to future generations. Protecting the world’s groundwater supply is paramount in a warming world and on continents that we now know are drying.

### Implications for sea level rise

The continents are now contributing more fresh water to GMSL rise than melting of the Greenland and Antarctic ice sheets. As the rate of continental drying and the intensity and frequency of extremes continue to increase, they may trigger unanticipated and frequent accelerations (*34*) in the rate of sea level rise that most coastal regions must better prepare for. Broader mitigation and adaptation strategies may involve storing more water on land or leaving more water in place, since increasing TWS would decrease rates of GMSL rise. Significantly slowing rates of global groundwater depletion, which, on its own, rivals GIC contributions (Fig. 6B, right), while facilitating large-scale groundwater recharge is, therefore, a global imperative, not only for preserving this precious resource for future generations but also for managing the global water balance to minimize groundwater depletion-driven freshwater inputs into the world oceans.

### Call to action

While efforts to slow climate change may be sputtering (*72*, *73*), there is no reason why efforts to slow rates of continental drying should do the same. Key management decisions and new policies, especially toward regional and national groundwater sustainability, and international efforts, toward global groundwater sustainability, can help preserve this precious resource for generations to come. Simultaneously, such actions will slow rates of sea level rise.

We hope that the findings of this work will serve to raise awareness of the urgent, global need to prepare for shrinking freshwater availability on land; greater vulnerability to sea level rise along coastal regions; and the interconnected, widespread impacts of continental drying on people, the environment, and the economy. Major coordinated, national, international, and global, transdisciplinary efforts are critically needed to elevate the level of awareness and action around continental drying and decreasing freshwater availability to that of the carbon cycle.

## MATERIALS AND METHODS

### GRACE/FO data

JPL GRACE/ GRACE-FO Mascon Release 6 Version 3 (JPL-M) (*74*) data are used to represent TWS anomalies. While mascon solutions from other processing centers are also available, the choice to use JPL-M stems from the lesser spatial correlation between the individual mascons. JPL-provided 1-sigma formal uncertainties in JPL-M are included in the uncertainty quantification described below. Global analysis is performed at the native resolution (~3° resolution fields provided at 0.5° × 0.5° grid for better delineation of the land-ocean boundaries) for the period from April 2002 to April 2024. The gap months between GRACE and GRACE-FO are ignored in the analysis while sporadic gaps in data records are interpolated after removing the climatology following the methods in previous studies (*21*).

### Trend and trend uncertainty computation

Long-term linear trends are computed by first removing the seasonal cycle by taking anomalies from the climatology and then applying an ordinary least-squares regression. Uncertainties on the trends aim to account for the uncertainty in GRACE JPL mascons and the uncertainty in the least-squares regression and are computed as follows: (i) Least-squares regression fit is computed along with the 90% confidence interval; (ii) the formal (1-sigma) uncertainty provided with the mascon product, which is unique to each mascon, is weighted by the area of that mascon within the region of interest. For example, if land covers only 25% of a mascon, then the error value for that mascon is multiplied by 0.25, and if the entire mascon is within land, then it is multiplied by 1. Then, root sum of squares for all the weighted error values is computed. These are the monthly uncertainties on the monthly TWS value for the region of interest; (iii) the average monthly formal (1-sigma) uncertainty is considered and applied as a positive uncertainty to the first half of the time series and as negative uncertainty to the second half of the time series, and vice versa. The trends computed from these provide the maximum and minimum regression values possible given the monthly formal uncertainty in data. The average difference from the regression fit with the min-max regression fits is considered as the uncertainty in long-term trends due to uncertainty in data; (iv) uncertainty obtained in step (iii) is added to the 90% confidence on the regression fit obtained in step (i). The total uncertainty, thus, is representative of the uncertainty in JPL mascons, as well as uncertainty in the regression model.

### Resolution enhancement for regional trends

The global analysis is performed at the original resolution of JPL-M. However, when discussing finer-scale trends such as grid-scale (Fig. 1) or boundaries with differing geographies, such as countries (Fig. 5) or smaller watersheds (fig. S5), it is useful to be able to infer sub-mascon-scale TWS information. Hence, for regional analysis, we enhance the resolution of the JPL-M to 0.25° × 0.25°. This is performed by bias correcting the higher-resolution (0.25° × 0.25°) TWS estimates from NASA Global Land Data Assimilation System Version 2 (GLDAS-2.2-DA) (*74*). The GLDAS-2.2-DA product is chosen because it assimilates, among other observation records with Catchment Land Surface Model L4 (CLSM4) (*75*), the GRACE/FO observations of the TWS [specifically, the mascon solutions from Center for Space Research; (*76*)], and, hence, its TWS output is already closer to GRACE observations than conventional, non-assimilated models. Still, biases arise due to the limitations in the land surface models in representing the cryospheric processes, subsurface hydrology, and human activities; the uncertainties in the forcing data; and the uncertainties in the assimilation process. Hence, a bias correction is applied as follows: (i) We first upscale the GLDAS model grid to a 0.5° grid matching with the JPL-M grid; (ii) to correct for the above bias, we apply a bias correction to the model output such that it matches the observed data at mascon scale. Because the variability of the fluxes within the extent of a mascon is unknown, we apply a mean correction over the entire extent of the mascon, i.e., we traverse across every mascon, and, for each one, we calculate the cumulative difference between the model data for that mascon. This value is normalized by the total number of 0.5° grids within the mascon, and the normalized bias is applied over every grid contained in the mascon; (iii) the normalized biases are resampled to 0.25° resolution through first-order spline interpolation using a widely used Python library (skimage) (*77*). (iv) Additionally, the biases are smoothed spatially using a moving-average filter of 9 × 9 grids to reduce the signal discontinuities at the mascon boundaries. This smoothed bias is added to the GLDAS output to get the final output that is the GLDAS model bias corrected with GRACE observations. Thus, the output retains the variability of the GLDAS model while being essentially unbiased at a global level and minimally biased at a mascon level relative to the GRACE/FO JPL mascons. The resolution enhancement is demonstrated for long-term trends over California in fig. S8. The model data are available starting February 2003 instead of April 2002 of GRACE; hence, the regional trends described in this study typically cover the period from February 2003 to April 2024, while the global analysis covers the period from April 2002 to April 2024.

### Identifying mega-drying regions

The mega-drying regions outlined in Fig. 1B were identified by grouping previously identified hot spots with interconnecting areas that have shown a change in trend from positive to negative, in particular since previous publications (*13*, *19*). Two criteria were used to define a mega-drying region: (i) a threshold value of −0.2 cm year^−1^, which is the 30th percentile of all the local trend magnitudes, was used to bound the mega-drying regions; and (ii) only those regions beyond the threshold that contained two or more previously identified drying hot spots. The grouping is subjective but is informed by experimentation and by the literature and is meant to highlight that vast, continuous regions between the previously studied hot spots are also drying, albeit at a moderate rate, and hence are typically overlooked. The Southern Hemisphere is largely excluded when identifying mega-drying regions because the threshold conditions are not typically met and also because, as we highlight in Fig. 4B, the region is typically governed by strong interannual variability.

### Robustness of trends

GRACE record is relatively short (~22 years against the recommended 30 or more) for climatological studies. The TWS trends over several regions, such as the Amazon, have flipped signs over the course of the GRACE mission. This begs the question, “How likely are the currently observed GRACE trends likely to persist?” To address this, we conduct a test for trend persistence by testing the sensitivity of the TWS trends to the increasing GRACE/FO record. As a starting step, local trends are computed for the first 5 years of the GRACE record. Then, the local trends are subsequently recomputed with addition of each successive month of GRACE/FO data. For example, the first iteration of local trend computation is from first 5 years of GRACE, and the past iteration of the trend computation is from the entire GRACE/FO record. This results in multiple (195) trend values for each location. The locations where the trend sign does not change for 95% of the instances are considered locations with robust drying or wetting trends and are shown in Fig. 4A. These are the locations where the trend signs did not change much (185 of the 195 instances), irrespective of when, during the GRACE record, the trends were assessed, and hence are likely to continue, unless the mechanisms governing the TWS trends change.

### Separating ocean, ice sheets, GIC, wetting, and drying regions

Ocean and land mascons are separated using the ancillary land-ocean mask associated with the mascon data product. The GIC mask (fig. S1) isolates masons that predominantly show ice mass changes (>1% of area is covered by glacier/ice), consistent with what has been used in previous publications (*30*). To be conservative in our analysis, mascons that neighbor those with strong ice mass trends (mascon IDs 97, 215, 216, 217, 269, 441, 3995, and 4071) are additionally included in the GIC mask to capture any residual ice mass leakage signals that exist. The wetting and drying regions are separated on the basis of the sign of their above-described long-term trends (fig. S7).

### Identifying and mapping growth of wetting and drying anomalies and extremes

To identify dry and wet anomalies, the TWS grids are first de-seasoned by subtracting the monthly climatology from each corresponding month to get deviations from the seasonal cycle, and the long-term mean is also removed in this process. Then, for each month, the locations where the anomalies are dry (and wet) are identified. The areas of those grids are computed and summed together to get total global area under wet and dry anomalies, and their trends are computed and provided in Table 2. Area under wet and dry anomalies is also computed after removing the GIC grids using the above-described mask. To identify wet and dry extremes, the local TWS data are first de-seasoned by removing the monthly climatology and then standardized by dividing them by their local SD. Any residual that is larger than a 1-sigma anomaly relative to climatology gets categorized as “extreme.” In other words, the locations where the monthly anomalies are greater than 1 are considered wet extremes and less than 1 are considered dry extremes. The areas of these grids are computed and summed together and provided as areas under wet and dry extremes, both with and without glaciers, in Table 2. To compute the timing and presence of wet and dry extremes in Fig. 3, the above-described monthly grids with extreme values are chunked into 5-year periods, and, for each grid, the number of months in these 5 years (totaling 60 months) when the grid showed an extreme value is recorded and converted to a percentage. For figs. S2 and S3, instead of 5-year periods, the monthly grids are aggregated into yearly grids, and the same statistic is calculated.

### Decomposing TWS into its SWE, surface water, soil moisture, and groundwater components

The trend is global non-glaciated TWS is further decomposed into TWS components groundwater, soil moisture, surface water, and snow water equivalent. For groundwater and soil moisture, GRACE-assimilated GLDAS-2.2-DA (the same model used for enhancing the resolution of TWS in the study) is used. Because surface water component is absent in GLDAS, it is obtained from WaterGAP 2.2d (*34*, *78*), which has been calibrated with observed river discharge and soil moisture worldwide, which generally yields much improved estimates of surface hydrological fluxes and storage compared to other global hydrological models. To avoid double counting of surface water, the equivalent value (SW_WG_) is removed from the soil moisture component from GLDAS (SM_GLDAS_). The SWE component from GLDAS showed a positive trend in the global SWE, which differed from most datasets. Hence, WaterGAP SWE was used instead. Thus, a new TWS is computed by adding the merged components

$${\text{TWS}}_{\text{merged}}={\text{GW}}_{\text{GLDAS}}+{\text{SW}}_{\text{WG}}+{\text{SWE}}_{\text{WG}}+\text{SM}$$

Here, SM = $\left({\text{SM}}_{\text{GLDAS}}-{\text{SW}}_{\text{WG}}\right).$ Contribution of the trends in individual components to the merged TWS is computed after removing the glaciated regions. The TWS signal within the Caspian Sea perimeter, which is missing in the models, is obtained from GRACE and is attributed as surface water. We acknowledge that the model outputs have uncertainties due to the limited representation of hydrology and the choice of forcing datasets and attempt to reduce the uncertainties by choosing a model that’s assimilated with GRACE for groundwater and soil moisture estimates and by choosing a model that’s calibrated against river discharge for surface water storage estimates. To reduce the uncertainties and biases caused by differing heterogeneity in model and observations, we also limit the TWS decomposition to the (non-glaciated) global scale.

### Annual renewable water

For the analysis illustrated in fig. S5, annual renewable water is considered as precipitation minus evapotranspiration minus environmental flows. The mean annual precipitation and evapotranspiration are computed from ERA5 data (*79*) overlapping the study period. The environmental flow data are based on (*70*).
