# Supplementary material for: Unprecedented continental drying, shrinking freshwater availability, and increasing land contributions to sea level rise
Source: Sci Adv. 2025 Jul 25;11(30):eadx0298. doi: 10.1126/sciadv.adx0298 (PMC12292907; doi:10.1126/sciadv.adx0298)
Supplement: Supplementary file 1 — Figs. S1 to S8 Tables S1 and S2 [file sciadv.adx0298_sm.pdf]

Science Advances
AAAS

Supplementary Materials for

Unprecedented continental drying, shrinking freshwater availability, and increasing land contributions to sea level rise

Hrishikesh A. Chandanpurkar *et al.*

Corresponding author: James S. Famiglietti, jay.famiglietti@asu.edu

Sci. Adv. **11**, eadx0298 (2025)
DOI: 10.1126/sciadv.adx0298

This PDF file includes:

Figs. S1 to S8
Tables S1 and S2

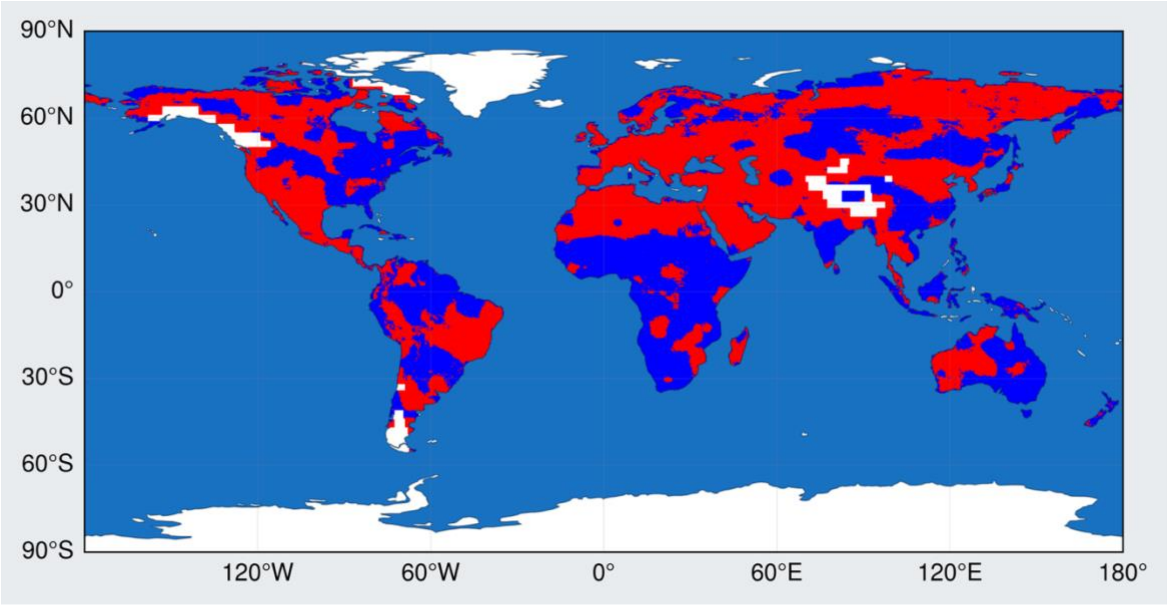

Fig. S1. Template used to mask glaciers and ice caps on land (GIC) and for computing global wetting and drying trends outside of GIC regions. Wetting (positive, blue) and drying (negative, red) TWS trends in the non-glaciated land areas from Figure 1A. Glaciers and ice sheets are shown in White.

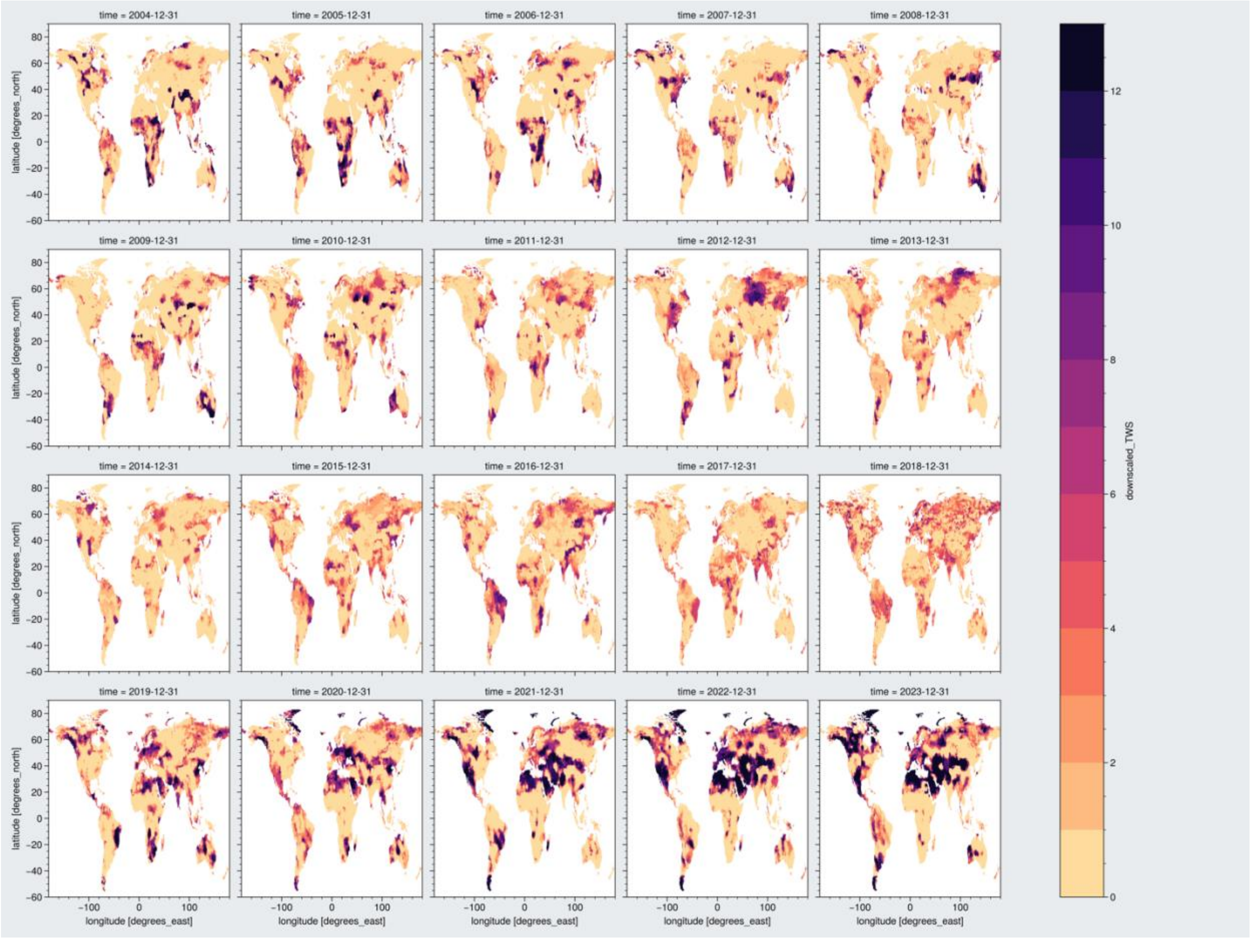

Fig. S2. Annual maps of the number of months each year of extreme TWS drying anomalies.

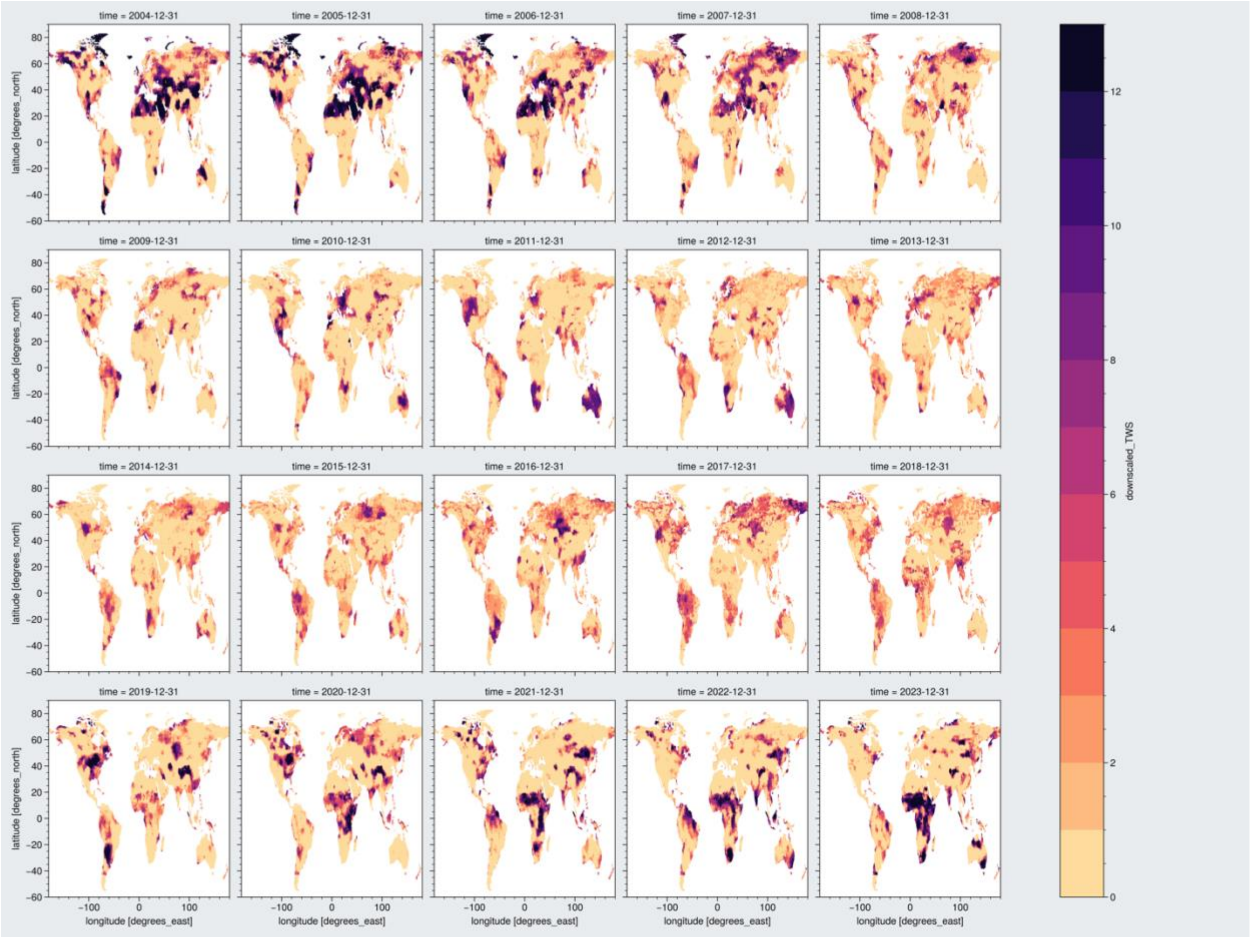

Fig. S3. Annual maps of the number of months each year of extreme TWS wetting anomalies.

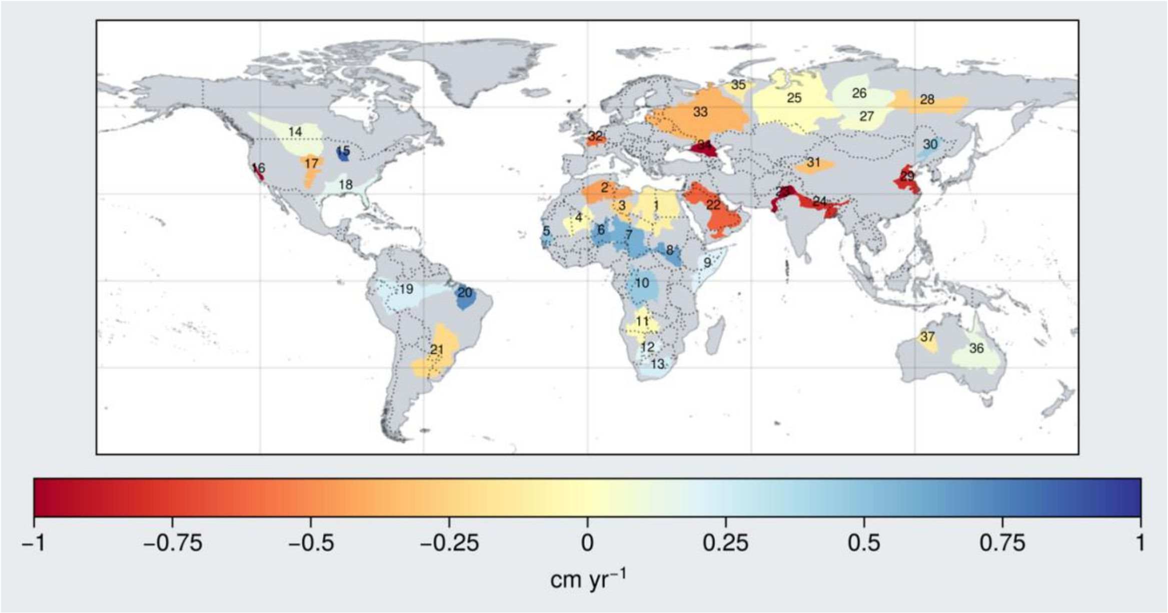

Fig. S4. Groundwater depletion in the world's major aquifers. TWS trends in 37 major aquifers of the world. Updated from figure 1 in (14) (CC BY-NC-ND 4.0; https://creativecommons.org/licenses/by-nc-nd/4.0/).

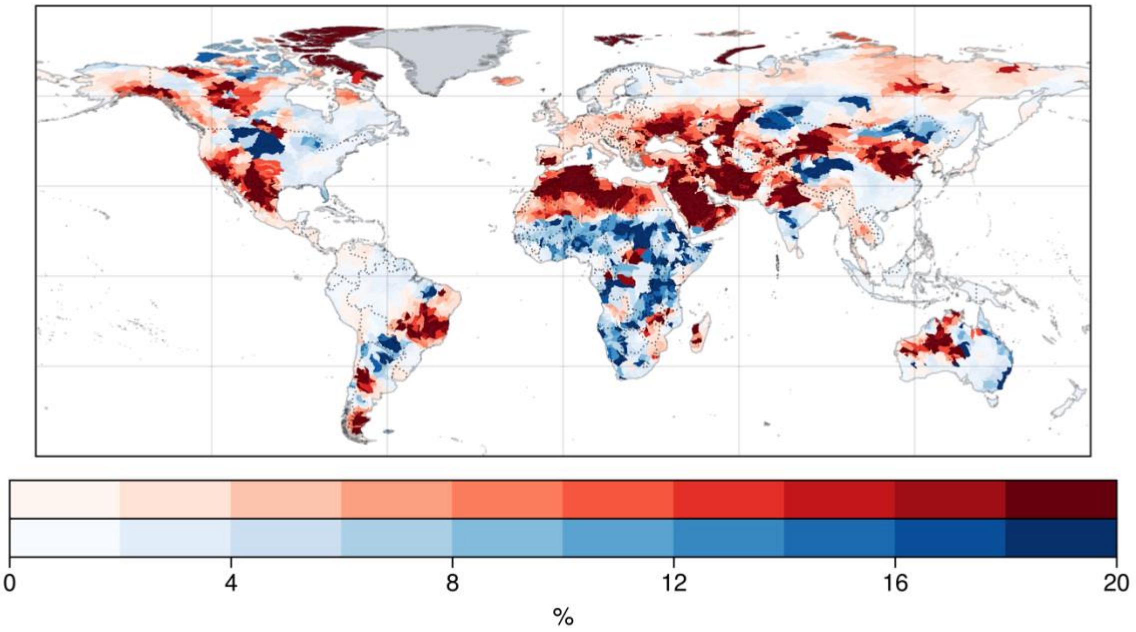

Fig. S5. Importance of the TWS trends. The TWS trend magnitude is presented as a percentage of annual renewable freshwater supply at level 5 watersheds. The colors indicate whether the basin TWS trend is negative (red) or positive (blue).

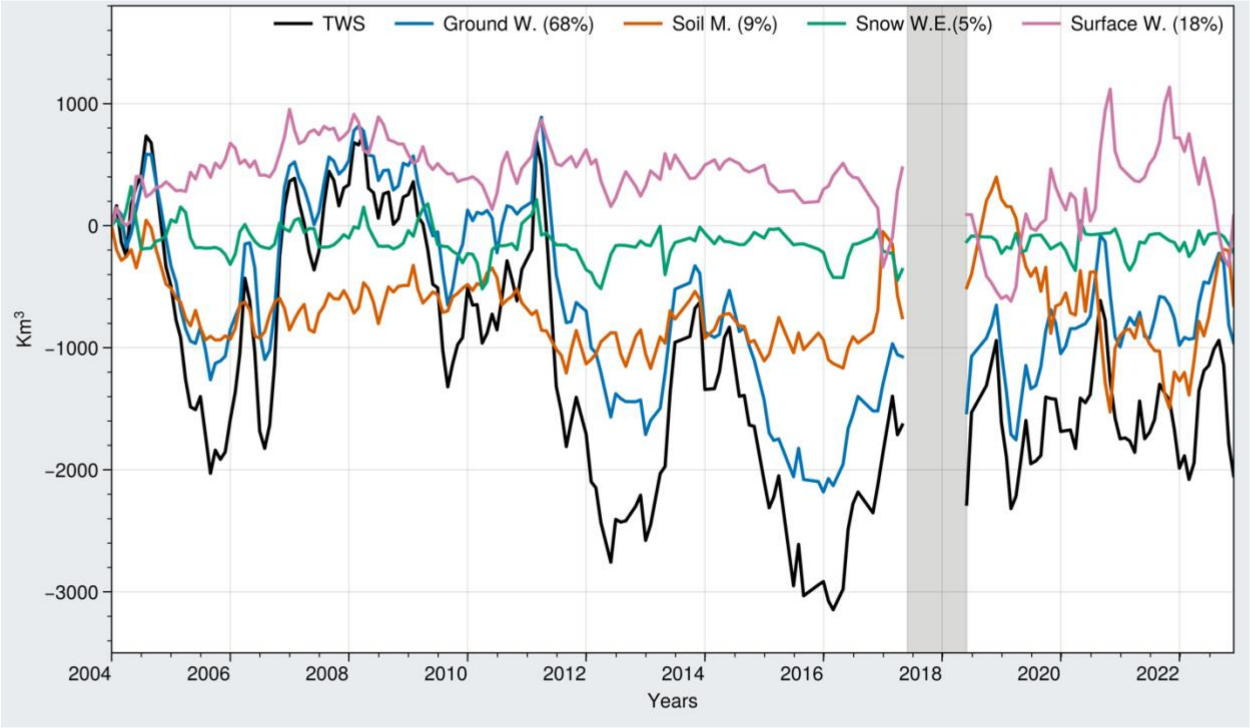

Fig. S6. The global TWS time series decomposed into snow water equivalent, surface water, soil moisture and groundwater components. A global hydrological model (71) was used to estimate the fraction of TWS change attributable to snow, surface water, soil moisture and groundwater for the time period 1/2004 through 12/2024.

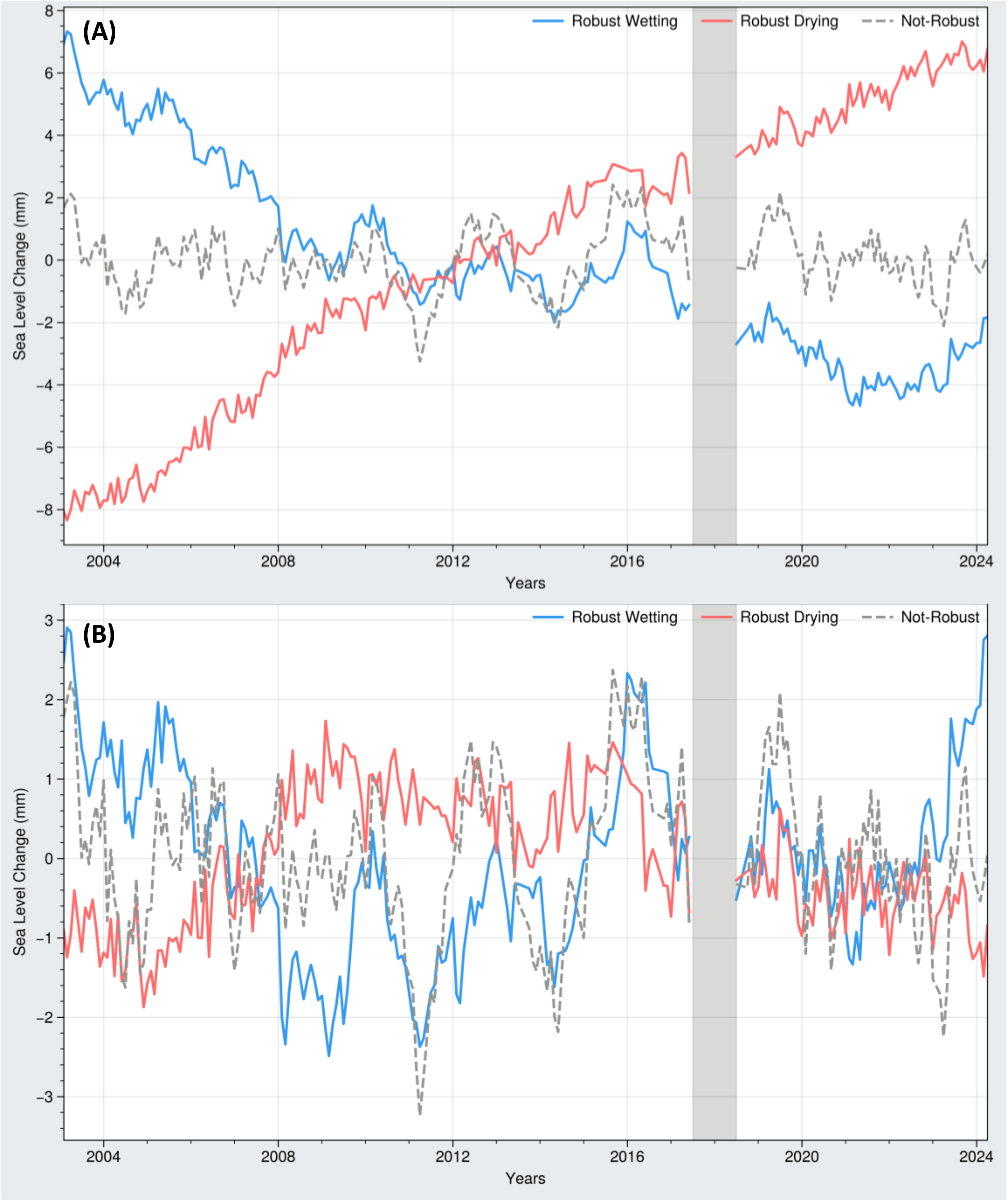

Fig. S7. Robust contribution from non-glaciated land regions to Global Mean Sea Level (GMSL). (A) Deseasoned time series from robust drying, robust wetting, and non-robust change from non-glaciated regions highlighted in Figure 4A. The time series are inverted to denote contribution to GMSL. (B) The same time series in (A) but de-trended to illustrate interannual variability.

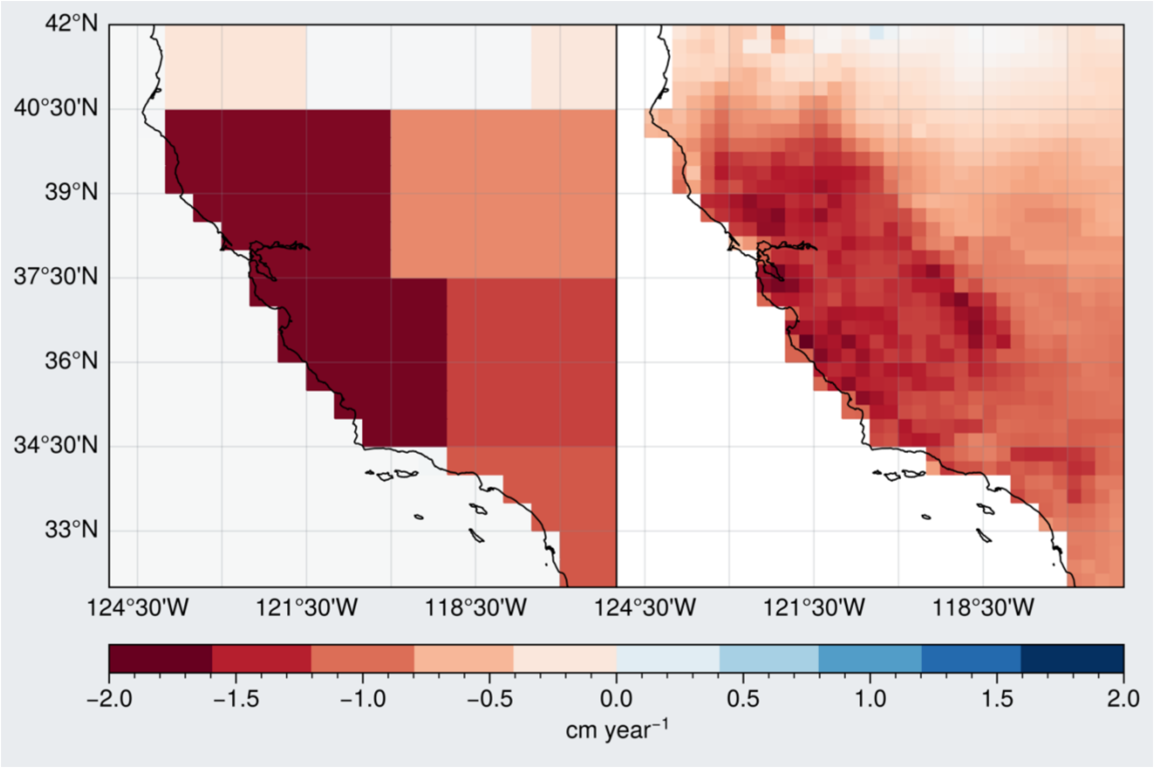

Fig. S8. Enhancing resolution of GRACE/FO data. A demonstration of resolution improvement over the native GRACE JPL Mascon resolution (left) by the approach used in this study (right). The figure shows long-term TWS trends over California.

Aquifer ID	Aquifer	TWS Trend (cm yr^{-1}). In brackets: total TWS trends if GICs are present.
1	Nubian Aquifer System (NAS)	-0.1± 0.01
2	Northwestern Sahara Aquifer System (NWSAS)	-0.45± 0.01
3	Murzuk-Djado Basin	-0.25± 0.01
4	Taoudeni-Tanezrouft Basin	-0.02± 0.01
5	Senegalo-Mauritanian Basin	0.5± 0.04
6	Iullemeden-Irhazer Aquifer System	0.61± 0.03
7	Lake Chad Basin	0.58± 0.05
8	Sudd Basin (Umm Ruwaba Aquifer)	0.65± 0.08
9	Ogaden-Juba Basin	0.2± 0.03
10	Congo Basin	0.46± 0.07
11	Upper Kalahari-Cuvelai-Upper Zambezi Basin	-0.03± 0.16
12	Lower Kalahari-Stampriet Basin	0.19± 0.04
13	Karoo Basin	0.24± 0.05
14	Northern Great Plains Aquifer	0.08± 0.08
15	Cambro-Ordovician Aquifer System	0.85± 0.14
16	Californian Central Valley Aquifer System	-1.49± 0.15
17	Ogallala Aquifer (High Plains)	-0.31± 0.08
18	Atlantic and Gulf Coastal Plains Aquifer	0.18± 0.1
19	Amazon Basin	0.23± 0.13
20	Maranhao Basin	0.7± 0.2
21	Guarani Aquifer System	-0.21± 0.13
22	Arabian Aquifer System	-0.64± 0.01
23	Indus Basin	-1.23± 0.07
24	Ganges-Brahmaputra Basin	-1.09± 0.09 (-1.4± 0.09)
25	West Siberian Basin	-0.03± 0.05
26	Tunguss Basin	0.06± 0.05
27	Angara-Lena Basin	0.06± 0.06
28	Yakut Basin	-0.26± 0.05
29	North China Aquifer System	-0.82± 0.1
30	Song-Liao Basin	0.49± 0.08
31	Tarim Basin	-0.39± 0.01 (-0.52± 0.02)
32	Paris Basin	-0.6± 0.07
33	Russian Platform Basins	-0.34± 0.05
34	North Caucasus Basin	-1.25± 0.06
35	Pechora Basin	-0.12± 0.06
36	Great Artesian Basin	0.12± 0.06
37	Canning Basin	-0.13± 0.1

Table S1. TWS trends (cm yr-1) for the major aquifer basins. The uncertainties represent 90% Confidence intervals on trend estimation. Updated from (14).

<table>
<tr><th>Data</th><th>Source URL</th></tr>
<tr><td>Terrestrial Water Storage;
1-sigma uncertainties;
mascon locations; land-ocean mask</td><td>https://podaac.jpl.nasa.gov/dataset/TELLUS_GRAC-GRFO_MASCON_CRI_GRID_RL06.1_V3</td></tr>
<tr><td>Higher-resolution model TWS and TWS components</td><td>https://disc.gsfc.nasa.gov/datasets/GLDAS_CLSM025_DA1_D_2.2/summary</td></tr>
<tr><td>Gridded Population</td><td>https://beta.sedac.ciesin.columbia.edu/data/set/gpw-v4-population-density-rev10</td></tr>
<tr><td>Precipitation; Evapotranspiration</td><td>https://cds.climate.copernicus.eu/datasets/reanalysis-era5-single-levels-monthly-means?tab=overview</td></tr>
<tr><td>Environmental flows</td><td>https://data.mendeley.com/datasets/n97vjpxhj2/1</td></tr>
<tr><td>ENSO time series</td><td>https://psl.noaa.gov/enso/mei/</td></tr>
<tr><td>Mountain glaciers and ice cap mask</td><td rowspan="5">https://zenodo.org/records/15487968</td></tr>
<tr><td>Enhanced resolution, bias-corrected, TWS trend map shown in Figure 1</td></tr>
<tr><td>Four drying mega-regions outlined in Figure 1</td></tr>
<tr><td>Robust drying and wetting trends (Figure 4a)</td></tr>
<tr><td>Ratio of long-term variance to interannual variance</td></tr>
</table>

Table S2: Sources of data used in this work.
